# RETRACTION: The Natural Carotenoid Crocetin and the Synthetic Tellurium Compound AS101 Protect the Ovary against Cyclophosphamide by Modulating SIRT1 and Mitochondrial Markers

**DOI:** 10.1155/omcl/9820439

**Published:** 2026-10-20

**Authors:** Oxidative Medicine and Cellular Longevity

RETRACTION: G. Di Emidio, G. Rossi, I. Bonomo, et al., “The Natural Carotenoid Crocetin and the Synthetic Tellurium Compound AS101 Protect the Ovary against Cyclophosphamide by Modulating SIRT1 and Mitochondrial Markers,” *Oxidative Medicine and Cellular Longevity*, 2017, 8928604, https://doi.org/10.1155/2017/8928604.

The above article, published online on 15 November 2017 in Wiley Online Library (http://wileyonlinelibrary.com), has been retracted by agreement between the Journal Editor‐in‐Chief, Jeannette Vasquez Vivar; and John Wiley & Sons Ltd. The retraction has been agreed due to concerns that have been raised directly and reported on PubPeer [1], regarding the Western blots shown in multiple figures. Specifcally:

• In Figure 3c, the first and third pAKT bands appear identical
• In Figure 3c, the first three ß‐actin bands appear identical to the first three ß‐actin bands shown in Figure 6b after being horizontally flipped and vertically compressed
• There is evidence of undeclared splicing in the Western Blots presented in multiple figures

The authors provided a response to the concerns, however after assessment by the editorial board, the results and conclusions can no longer be considered reliable. The article is therefore retracted.

The authors disagree with the retraction.
